# Successful multiple-exchange peritoneal dialysis in a patient with severe hematological toxicity by methotrexate: case report and literature review

**DOI:** 10.1590/2175-8239-JBN-2018-0095

**Published:** 2018-09-21

**Authors:** Arbey Aristizabal-Alzate, John Fredy Nieto-Rios, Catalina Ocampo-Kohn, Lina Maria Serna-Higuita, Diana Carolina Bello-Marquez, Gustavo Adolfo Zuluaga-Valencia

**Affiliations:** 1 Hospital Pablo Tobón Uribe MedellínAntioquia Colombia Hospital Pablo Tobón Uribe, Medellín, Antioquia, Colombia.; 2 Universidad de Antioquia MedellínAntioquia Colombia Universidad de Antioquia, Medellín, Antioquia, Colombia.; 3 University of Tüebingen Tübingen Germany University of Tüebingen, Tübingen, Germany.

**Keywords:** Methotrexate, Pancytopenia, Renal Insufficiency, Peritoneal Dialysis

## Abstract

Methotrexate is an effective medication to control several diseases; however, it can be very toxic, being myelosuppression one of its main adverse effects, which increases in severity and frequency in patients with renal failure. We present the case of a 68-year-old man with chronic, end-stage renal disease associated with ANCA vasculitis, under treatment with peritoneal dialysis, who received the medication at a low dose, indicated by disease activity, which presented as a complication with severe pancytopenia with mucositis that improved with support measures and multiple-exchange peritoneal dialysis. We reviewed 20 cases published to date of pancytopenia associated with methotrexate in patients on dialysis and found high morbidity and mortality, which is why its use in this type of patient is not recommended. However, when this complication occurs, a therapeutic option could be the use of multiple-exchange peritoneal dialysis in addition to supportive therapy for drug-related toxicity, although it is recognized that studies are required to show the role of multiple-exchange peritoneal dialysis in the removal of this medication.

## INTRODUCTION

Methotrexate is a disease-modifying, anti-rheumatic drug, used in treatment schemes for different diseases; however, its long-term use can cause adverse effects in up to 61% of patients, causing the discontinuation of medication in 20%^1^. One of the most feared complications due to its high morbidity and mortality is myelosuppression;^2^^,^^3^ impaired renal function, advanced age, diabetes mellitus, folic acid deficiency, and hypoalbuminemia are the main risk factors for developing the complication.^4^

Despite the use of methotrexate for over 20 years, there is no clear indication about the safety of this drug in patients with end-stage renal disease (ESRD) or renal replacement therapy (hemodialysis or peritoneal dialysis). The guidelines published by the American College of Rheumatology^5^ and the manufacturer's information^6^ are not specific regarding the safety of administering methotrexate in the presence of established ESRD and/or dialysis, and its use at low doses in these patients is still controversial. This gap may result in the false belief that its administration is safe in such circumstances; in addition, some authors have interpreted that bone marrow depression caused by methotrexate as an idiosyncratic effect.^6^ However, there are reports of patients with ESRD who after receiving methotrexate therapy, even at low doses, presented severe complications, and currently there is no clarity about which is the best dialysis therapy to eliminate this toxic substance.

Next, we report the case of a patient diagnosed with ESRD on treatment with peritoneal dialysis who was treated with methotrexate for a diagnosis of granulomatosis with polyangiitis (Wegener). A few days after starting the medication, the patient developed a severe pancytopenia, which was successfully handled with support therapy and intensive peritoneal dialysis.

## CASE REPORT

A 68-year-old man presented with a diagnosis of granulomatosis with polyangiitis that evolved for 6 years with paranasal, ocular, pulmonary, and renal involvement. As a consequence of ESRD, the patient required automated peritoneal dialysis for the last 3 years. For relapse of vasculitis, he received 7.5 mg methotrexate weekly. After the second dose, he presented odynophagia, asthenia, adynamia, melenic depositions, bleeding from the gums, oral ulcers, and painful lesions on the skin of his lower limbs. On admission, it was found a sleepy, febrile patient, with blood pressure 99/57 mmHg, heart rate of 97 per minute, and respiratory rate of 17 per minute. Clinical findings included necrotic lesions on the lower lip, ulcers on the cheeks, aphthae on the tongue, and macules with erythematous edges on his inner thighs. Laboratory tests showed high levels of inflammatory reactants, pancytopenia, and elevated levels of methotrexate (table 1). With the above, it was established a diagnosis of methotrexate toxicity with hematological compromise, as well as of skin and mucous membranes lesions; and as a consequence, febrile neutropenia with septic shock was diagnosed. Management was started in the intensive care unit with piperacillin, tazobactam, vancomycin, fluconazole, steroids in stress doses, support with norepinephrine, calcium folinate, and granulocyte colony stimulating factor. He was also managed with multiple-exchange peritoneal dialysis 20 hours a day (2 periods of 10 hours, 11 liters per period distributed in 6 liters x 2.5% and 5 liters x 1.5%, 10 cycles in total) in order to increase the clearance of the methotrexate and avoid the installation of a hemodialysis catheter in a patient with severe thrombocytopenia. All the cultures taken were negative, so antibiotics were suspended after 5 days. The patient evolved towards improvement and within a week, his hematological cell count recovered, fever did not return, and he recovered from his skin and mucosal lesions; in addition, a new measurement of methotrexate showed no detectable levels. 

**Table 1:** Laboratory tests

Study	Laboratory values at admission	Laboratory values at discharge
BUN	78 mg/dL	
C-Reactive protein	22.78 mg/dL	
Erythrosedimentation	120 mm/hour	
Hemogram	Hemoglobin: 8.6 g/dL, Leukocytes 1300 x mm^3^, Neutrophils: 14 %, Lymphocytes: 63%, eosinophils: 11%, Platelets: 26,000 x mm^3^	Hemoglobin 7.7 g/dL Leukocytes 13,300 x mm^3^, Neutrophils 70 %, Lymphocytes 19%, Platelets 222,000 x mm^3^.
Ionogram	Na: 138 mmol/L, Cl 96 mmol/L, K 3.6 mmol/L, Ca 7 mg/dL.	
Blood cultures	Negative	
Gram and sputum culture	Negative	
Urine culture	Negative	
Methotrexate levels	0.5 micromoles/L	Undetectable
Chest x-ray:	Basal bilateral mixed pulmonary infiltrates.	
Computerized Axial Tomography of the chest	Alveolar opacities in the lower lobes and posterior segment of the right upper lobe.	

## DISCUSSION

Methotrexate (molecular weight 454.4 kDa) is an antimetabolite of folic acid that inhibits dihydrofolate reductase, a key enzyme for the production of tetrahydrofolates, which are required for the synthesis of purines and pyrimidines, and therefore, it inhibits the cell cycle.^7^

The pharmacokinetics of methotrexate is highly variable and unpredictable.^8^ It is rapidly absorbed after oral administration, reaching serum levels after one hour, with a binding to albumin of 50%. It is distributed in extravascular compartments, including kidneys, liver, and synovial fluid. Within the cells, it is converted to methotrexate polyglutamates, which are more potent increasing its intracellular half-life. It is partially oxidized in the liver to 7-hydroxy-methotrexate, a less active metabolite, and it is excreted mainly by the kidneys, and in lesser amount by the liver. More than 90% of the absorbed amount is excreted in the urine at 48 hours by glomerular filtration and active tubular secretion. The elimination half-life is 5 to 8 hours, but it increases significantly when there is acute or chronic renal failure, especially in advanced stages, which leads to a reduction in the clearance of methotrexate and an increase in its toxicity.^9^ At present, there are no pharmacokinetic parameters that correlate with efficacy, and plasma concentration cannot predict clinical response or adverse effects.^10^

Methotrexate is widely used in cancer, rheumatological diseases, inflammatory bowel disease, and obstetric conditions. In patients with rheumatoid arthritis and normal renal function, the recommended dose is between 5 and 7.5 mg week, with a maximum dose of 15 mg week.^11^ This drug mainly affects the cells of fast division, a reason why it has deeper action on the cells of the bone marrow and the gastrointestinal tract. The destruction of bone marrow cells predisposes the patient to thrombocytopenia, granulocytopenia, and lymphopenia, which can lead to life-threatening infections, severe anemia and gastrointestinal tract hemorrhage. Other additional adverse effects are nausea, vomiting, stomatitis, and pulmonary and hepatic toxicity.

Toxicity associated with the use of methotrexate has been reported even in therapeutic doses and in patients who don't need renal replacement therapy. Gutierrez-Ureña and colleagues identified 70 dialysis-free patients who presented pancytopenia related to methotrexate, with a mortality rate of 17.1%.^4^ Lim and colleagues identified 25 cases in a period of 5 years, with an average age of 76 years, a dose of 12.5 mg week, a therapeutic duration of 36 months, 40% with a leukocyte count less than 2000/mm³, 30% (8 out of 25) of the patients had renal failure, and the mortality rate was 28%.^3^ In the presence of advanced renal failure and in patients on dialysis, the risk of toxicity is greater, even at low doses, with presence of higher plasma levels and longer half-lives (even detectable up to 3 weeks after receiving small doses of 2.5 mg).

The literature reports several cases of pancytopenia in patients on dialysis after therapy with low doses of methotrexate, even with intralesional application; these cases were published between 1990 and 2017.^11^^,^^25^ Liu WC and colleagues reported 1 case and collected another 15 cases from 12 publications in the literature;^15^ we found another 3 cases published,^22^^,^^24^ and with this case report, we have a total of 20 cases reported with diagnosis of pancytopenia associated with methotrexate in patients on dialysis (see table 2).

**Table 2:** Clinical data of patients on dialysis with myelosuppression induced by methotrexate

Reference	Sex	Age	Type of dialysis	Indication	Dose (mg/week)	Duration (weeks)	Cumulative Dose (mg)	Level of methotrexate (ᵘmol/L)	Nadir leukocytes	Result
Diskin ^(^12^)^	M	60	PD	RA	10	2	20	0.53	300	Dead
Chess ^(^13^)^	M	64	PD	Psoriasis	-	-	35	-	300	Dead
Sun ^(^14^)^	F	33	PD	Lupus Arthritis	5.0	2	25	-	600	Recovered
Liu WC ^(^15^)^	F	61	PD	Eczema	7.5	3	22.5	0.08	30	Recovered
Elman ^(^16^)^	F	52	HD	RA	2.5	Single dose	2.5	0.13	500	Dead
Elman ^(^16^)^	F	47	HD	Systemic sclerosis	2.5	Single dose	2.5	-	1500	Recovered
Nakamura ^(^17^)^	M	57	HD	RA	5.0	Single dose	5.0	0.03	100	Recovered
Chatham ^(^18^)^	M	49	HD	Myositis	-	2	-	-	90	Recovered
Chatham ^(^18^)^	M	52	HD	Myositis	5.0	Single dose	5.0	-	2200	Recovered
Chatham ^(^19^)^	F	61	HD	Psoriasis	2.5	3	7.5	-	50	Dead
Boulanger ^(^11^)^	F	60	HD	RA	5.0	2	10.0	-	1300	Recovered
Basile ^(^19^)^^(^20^)^	F	74	HD	RA	5.0	2	10.0	-	1700	Recovered
Boey ^(^9^)^	M	66	HD	Psoriasis	5.0	2	10.0	0.03	70	Recovered
Yang ^(^20^)^	F	55	HD	RA	7.5	12	90.0	0.11	400	Dead
Seneschal ^(^21^)^	M	76	HD	PB	5.0	1.5	7.5	0.47	550	Dead
Cheung ^(^22^)^	M	56	HD	Psoriasis	2.5	Single dose	2.5	0.06	60	Dead
Liu H. ^(^23^)^	M	48	HD	PB	10	2	20	0.14	1800	Recovered
Mima ^(^24^)^	M	46	HD	RA	-	-	-	-	690	Recovered
Flynn ^(^25^)^	M	68	PD	CA squamous cells	2.5 intra-lesion	Single dose	25	0.03	600	Recovered
Current report	M	68	PD	vasculitis	7.5	2	15	0.005	600	Recovered

M: Male, F: Female, PD: Peritoneal Dialysis, HD: Hemodialysis, RA: Rheumatoid Arthritis, PB: Pemphigoid Bullous, CA: Cancer; modified table of the article published by Liu WC, Chen HC, Chen JS. Clinical dilemma over low-dose methotrexate therapy in dialysis patients: a case report and review of literature. Iran J Kidney Dis. 2014; 8 (1): 81-4 (15).

When analyzing these 20 patients, average age of 57.6 years, it is found that they received a cumulative dose of 17.5 mg. The average dose was 5.2 mg/week, for an average therapeutic duration of 2 weeks. Nineteen of the 20 patients had nadir leukocytes less than 2000/mm³, which is defined as severe leukopenia. The mortality rate was 35% (7 of 20). Comparing patients who received methotrexate without dialysis with patients on dialysis, it was found that dialysis patients were relatively younger, had a short duration of dose, low cumulative dose, and severe leukopenia compared to patients who were not on dialysis. Mortality was also higher in dialysis patients. In general, the patients who died had a lower nadir of leukocytes and higher levels of methotrexate than those who recovered. In any case, the final result depends on several factors, such as comorbidities, cumulative dose, methotrexate clearance, early detection and medical care.

In the case reported here, the patient was on peritoneal dialysis and a low dose of methotrexate was used according to the indication for rheumatological pathology. As he presented a complication of severe pancytopenia and mucositis, he was possibly more susceptible to presenting toxicity for this drug.

The efficacy of methotrexate removal by dialysis has been associated with the toxicity of methotrexate. There are reports of methotrexate removal by hemodialysis and hemoperfusion, with both methods effectively removing the drug thanks to its binding to proteins by 50%. However, there is a post-dialysis rebound in the methotrexate concentration of 90-100% to the levels prior to the procedure; therefore, patients may require daily or continuous renal replacement therapy to avoid rebound toxicity.^25^ However, little is known about removal by peritoneal dialysis.

Ahmad and colleagues^26^ reported a case of acute kidney injury caused by methotrexate, in which emergency acute peritoneal dialysis was performed for 7 days, using 34 to 40 L/day of dialysate without finding a change in the serum concentration of this drug; however, the number or frequency of exchanges or volumes of permanence were not reported. Diskin and colleagues^12^ reported that clearance of methotrexate in peritoneal dialysis was less effective than in hemodialysis; they performed an exchange of peritoneal dialysis (3 L volume for 6 hours) followed by high flow hemodialysis for 7 hours. After the 6-hour peritoneal dialysis exchange, the methotrexate concentration only decreased from 0.53 to 0.47 micromoles/liter. They also showed that the removal of methotrexate by means of peritoneal dialysis was mainly in the first hour of exchange. However, Murashima and colleagues^27^ reported the case of a patient on peritoneal dialysis treated with a high dose of methotrexate for lymphoma in the central nervous system. For the initial cycles of methotrexate, he received temporary high-flux daily hemodialysis, starting 24 hours after the infusion of methotrexate to avoid toxicity; but later, due to problems with vascular access, he was treated with continuous peritoneal dialysis of multiple exchanges for the last 2 cycles of chemotherapy (the intensive peritoneal dialysis consisted of 20 cycles in 24 hours, with residence times of 30 minutes, 1.8 liters of fluid admitted). The clearance for high flow hemodialysis was 0.77 mL/kg/min; and for peritoneal dialysis, 0.65 mL/kg/min. Despite a lower clearance for peritoneal dialysis, the patient did not develop clinical evidence of methotrexate toxicity.

In our case, the use of multiple-exchange peritoneal dialysis was chosen to avoid the installation of a hemodialysis catheter due to thrombocytopenia. It was adjusted to the automated peritoneal dialysis prescription that the patient received previously; basically, the night cycle of automated peritoneal dialysis was repeated during the day, thus increasing the volume of peritoneal dialysis from 11 liters to 22 liters. A new measurement of methotrexate showed no detectable levels, the patient recovered the leukocyte count, and survived; therefore, we propose that an option for the treatment of methotrexate-associated toxicity in patients on peritoneal dialysis could be multiple-exchange peritoneal dialysis without having to transfer the patient to hemodialysis; in our case, this was easily accomplished by modifying the usual peritoneal dialysis of the patient. However, more studies are required to confirm if such dialysis method can prevent and correct the toxicity associated with methotrexate.

## CONCLUSION

In conclusion, we do not recommend the use of methotrexate in patients with advanced renal failure or who are on dialysis. If its use is indispensable, we recommend a close clinical and para-clinical follow-up to detect the toxicity early. In the presence of methotrexate toxicity, multiple-exchange peritoneal dialysis may be an alternative treatment for selected patients.
